# Aggravated MRSA pneumonia secondary to influenza A virus infection is derived from decreased expression of IL‐1β

**DOI:** 10.1002/jmv.26329

**Published:** 2020-09-16

**Authors:** Yunfeng Shi, Xiaohan Shi, Jingjing Liang, Jinmei Luo, Junhui Ba, Jianning Chen, Benquan Wu

**Affiliations:** ^1^ Medical Intensive Care Unit, Department of Respiratory and Critical Care Medicine The Third Affiliated Hospital of Sun Yat‐Sen University Guangzhou China; ^2^ Department of Respiratory and Critical Care Medicine Institute of Respiratory Diseases of Sun Yat‐Sen University Guangzhou China; ^3^ Department of Emergency The Third Affiliated Hospital of Sun Yat‐Sen University Guangzhou China; ^4^ Department of Pathology The Third Affiliated Hospital of Sun Yat‐Sen University Guangzhou China

**Keywords:** interleukin‐1β, influenza A virus, methicillin‐resistant *Staphylococcus aureus*, NLRP3 inflammasome, secondary infection

## Abstract

Secondary methicillin‐resistant *Staphylococcus aureus* (MRSA) infection is a cause of severe pneumonia with high mortality during influenza A virus (IAV) pandemics. Alveolar macrophages (AMs) mount cellular defenses against IAV and MRSA infection, which occurs via the nucleotide‐binding domain‐like receptor protein 3 (NLRP3) inflammasome. However, the activity and function of the NLRP3 inflammasome in MRSA pneumonia secondary to IAV infection remain unclear. To clarify this, we studied MRSA infection secondary to IAV both in vitro and in mouse model. The expression of the NLRP3 inflammasome was evaluated by quantitative reverse transcription polymerase chain reaction, immunofluorescence, Western blot, and enzyme‐linked immunosorbent assay. The lung pathology and the rate of weight change were observed. We found that IAV infection for 1 week activated NLRP3 inflammasome. The enhanced expression of NLRP3, caspase‐1, and cleaved caspase‐1 was associated with MRSA infection secondary to IAV, but the expression of interleukin (IL)‐1β decreased in superinfection with MRSA both in vitro and in vivo. The aggravated inflammatory pathology in MRSA pneumonia secondary to IAV infection was associated with decreased expression of IL‐1β. And increased weight loss in MRSA pneumonia secondary to IAV infection was related to decreased concentration of IL‐1β in serum. It infers that superinfection with MRSA reduces expression of IL‐1β someway, and decreased expression of IL‐1β impairs the host immunity and leads to aggravated pneumonia. These results contributed to our understanding of the detailed activity of the NLRP3 inflammasome, IL‐1β, and their relationship with aggravation of MRSA pneumonia secondary to IAV infection. Immunotherapy targeting the IL‐1β signaling pathway could be possible therapeutic strategy for secondary MRSA pneumonia.

## Highlights

- MRSA pneumonia secondary to influenza A virus infection causes severe outcome.
- NLRP3 inflammasome plays a role in the pathogenesis of MRSA pneumonia secondary to IAV infection.
- The aggravated inflammatory pathology in MRSA pneumonia secondary to IAV infection was associated with decreased expression of IL‐1β.
- Immunotherapy targeting the NLRP3 inflammasome and IL‐1β signaling pathway could be possible therapeutic strategy for secondary MRSA pneumonia.

## 1 INTRODUCTION

The influenza virus is a member of the family Orthomyxoviridae, and can be classified into A, B, and C subtypes. Influenza A virus (IAV) infects type II epithelial cells and alveolar macrophages (AMs) in the airway and alveoli in humans, and causes acute respiratory infectious diseases including seasonal flu, bronchitis, and pneumonia. Approximately, 600 million people around the world suffer from IAV infections every year, and occasional pandemics cause greater threat to public health.^1^, ^2^ For most cases of IAV infection, the infection runs its course within 1 week, and the patient makes a full recovery. However, severe pneumonia and increased risk of mortality are sometimes caused by complications following secondary bacterial infection, which usually occurs about 1 week after IAV infection.^3^, ^4^, ^5^, ^6^, ^7^ *Staphylococcus aureus* (*S. aureus*) is one of the most frequent causes of secondary infection in patients with IAV, and secondary methicillin‐resistant *S. aureus* (MRSA) pneumonia is the most lethal secondary infection.^8^ ^‐^ ^10^ Because of the high risk and severity of secondary MRSA pneumonia, antibiotic treatment has been recommended in influenza pandemic preparedness strategy.^11^ However, because of the multiantibiotic resistance of MRSA, searching and applying immunotherapy for secondary MRSA pneumonia is of great necessity.^12^

As the major innate immunocytes in the respiratory tract, AMs initiate immune response against infections such as IAV and MRSA via pattern recognition receptors.^13^, ^14^ These include Toll‐like receptors (TLRs) and nucleotide‐binding oligomerization domain‐like receptors (NLRs). The latter can assemble with apoptosis‐associated speck‐like protein containing a caspase activation and recruitment domain (ASC), and caspase‐1 to form a multiprotein complex called the inflammasome.^15^

Being composed of nucleotide‐binding domain‐like receptor protein 3 (NLRP3), ASC, and caspase‐1, the NLRP3 inflammasome plays an important role in the host response to infectious disease. The activity of NLRP3 can be suppressed by NLRP3‐specific inhibitor MCC950.^16^ The activation of the NLRP3 inflammasome upon infection requires two signaling pathways.^17^, ^18^ Signaling pathway 1 is the priming of transcription and synthesis of NLRP3 and pro‐interleukin (IL)‐1β through the TLR‐MyD88‐NF‐κB pathway. The second signaling pathway is the formation of NLRP3 inflammasome, autocatalysis of caspase‐1 into cleaved caspase‐1, cleavage of pro‐IL‐1β into IL‐1β, and secretion of IL‐1β.^19^, ^20^ As an important immune signaling cytokine, IL‐1β initiates downstream inflammation through multiple mechanisms, including cytokine production and neutrophil recruitment.^21^

Research focused on the pathogenesis of *S. aureus* infection secondary to IAV has accumulated for these years.^9^, ^14^, ^18^, ^22^, ^23^, ^24^, ^25^, ^26^, ^27^, ^28^, ^29^ However, there is much less information available on MRSA pneumonia secondary to IAV infection.^30^ Blyth et al^10^ concluded that coinfection of IAV and bacteria synergistically aggravated inflammatory injury to the host. Deterioration of the lung has been observed to be enhanced by superinfection of MRSA and influenza.^22^, ^26^ However, the functions and mechanisms of the NLRP3 inflammasome in the severe MRSA pneumonia secondary to IAV infection remain unknown.

In the present study, the expression of the NLRP3 inflammasome was evaluated by quantitative reverse transcription polymerase chain reaction (RT‐qPCR), immunofluorescence, Western blot, and enzyme‐linked immunosorbent assay (ELISA), and the lung pathology and the rate of weight change were observed. We found that enhanced expression of NLRP3 and caspase‐1 during MRSA infection secondary to IAV, but the expression of IL‐1β decreased in MRSA infection secondary to IAV both in vitro and in vivo. The aggravated inflammatory pathology in MRSA pneumonia secondary to IAV infection was associated with decreased expression of IL‐1β. The rate of weight loss in infected mice negatively correlated with concentration of IL‐1β in serum. And increased weight loss in MRSA pneumonia secondary to IAV infection was related to decreased concentration of IL‐1β in serum. It infers that superinfection with MRSA reduces expression of IL‐1β someway, and decreased expression of IL‐1β impairs the host immunity and leads to aggravated pneumonia.

## 2 MATERIALS AND METHODS

### 2.1 Ethics statement

The experimental protocol containing euthanasia criteria was established according to ethical guidelines and was approved by the Experimental Animal Ethics Committee of the Third Affiliated Hospital of Sun Yat‐Sen University, China.

### 2.2 Influenza virus strain and titration

IAV H1N1, an IAV H1N1/FM1 mouse lung‐adapted strain, was provided by the Department of Microbiology and Immunology at the Basic Medical College, Jinan University, China. IAV H1N1 can infect murine macrophages and cause severe pneumonia in mice according to preliminary experiments.^31^, ^32^ After two rounds of routine chick embryo resuscitations, the virus concentrations causing 50% cell infection (TCID_50_) and 50% mouse mortality (LD_50_) were determined respectively. A 100 μL virus solution of 10 times the TCID_50_ concentration was added into each well containing murine macrophages, and 50 μL of virus solution at the LD_50_ concentration was given to each mouse.

### 2.3 MRSA strain and titration

The MRSA strain (ATCC 43300) was obtained from the Laboratory of Respiratory and Critical Medicine of the Third Affiliated Hospital of Sun Yat‐Sen University, Guangzhou. MRSA was cultivated on blood agar plates overnight, then suspended in phosphate‐buffered saline (PBS) and adjusted to an optical density at 600 nm (OD_600_) of 0.6, corresponding to 1 × 10^9^ CFU/mL. Tenfold the multiplicity of infection (MOI) of MRSA was applied in vitro to murine macrophages, and 50 μL MRSA suspension was applied in vivo in the mouse model.

### 2.4 Macrophage culture and stimulation in vitro

The murine macrophage cell line RAW264.7 was purchased from the Institute of Basic Medical Sciences at the Chinese Academy of Medical Sciences (Beijing, China). Cells were cultured on human fibronectin‐coated six‐well plates at a density of 1 × 10^6^ cells/well in Dulbecco modified Eagle's medium (Gibco, Gaithersburg, MD) supplemented with 10% fetal bovine serum (PAN, Germany), and grown in a 95% air and 5% carbon dioxide humidified atmosphere at 37°C. The viability of the cells measured by methylthiazolydiphenyl‐tetrazolium bromide assay in each group was more than 98%. The cells were stimulated with IAV H1N1 and MRSA as described below when the cell density reached 2 × 10^6^ cells/well. The cells were divided into the control, IAV, and IAV + MRSA groups. Aliquots of 100 μL virus solution were added to IAV and IAV + MRSA groups on day 0 (0 hours), and the same volume of virus culture medium was added to the control group. For all groups, culture medium was replaced on day 2 (48 hours), day 4 (96 hours), and day 6 (144 hours). For the IAV and IAV + MRSA groups, fresh virus solution was added each time after replacement of the culture medium. Subsequently, 10‐fold the MOI of MRSA in solution was added to the IAV + MRSA group on day 6 (144 hours), and the same volume of PBS was added to the control and IAV group. After 24 hours, the cells and supernatants were collected on day 7 (168 hours). Morphological changes to adherent cells were visualized with an Olympus phase‐contrast microscope in the culture medium (Olympus Optical Co., Ltd, Tokyo, Japan). Experiment with NLRP3 inhibitor MCC950 was performed as supplement. Except 7.5 μM MCC950 (Selleck Chemicals, Houston, TX) was applied to the IAV + MCC950 and IAV + MRSA + MCC950 group on day 6 (144 hours), and the same volume of PBS was added to the other groups. The protocol of cell culture and stimulation with IAV and MRSA was exactly the same as before.

### 2.5 Animal preparation and in vivo infection

C57BL/6 specific‐pathogen‐free (SPF) grade mice were purchased from the Medical Animal Experiment Center of Guangdong Province, animal license: SCXK (YUE) 2013‐002. Mouse breeding was carried out in an SPF environment with sufficient feed and drinking water, in a temperature‐controlled animal facility (temperature: 20 ± 2°C, humidity: 50%) with a 12‐hours diurnal cycle. Fifteen male C57BL/6 mice (6‐7 weeks old) were randomly divided into the control, IAV and IAV + MRSA group (n = 5/group). On day 0 (0 hours), mice in the control group were intranasally inoculated with 50 μL of PBS, and mice in the IAV and IAV + MRSA groups were intranasally inoculated with 50 μL of virus solution. On day 6 (144 hours), mice in the control and IAV groups were intranasally inoculated with 50 μL of PBS, and mice in the IAV + MRSA group were intranasally inoculated with 50 μL of MRSA suspension. On day 7 (168 hours), after blood was collected from the orbit, all mice were euthanized via cervical dislocation to harvest lungs. Anesthesia by intraperitoneal injection of chloral hydrate solution (10%, 80 μL) was performed on each mouse before intranasal inoculation. Anesthesia by intraperitoneal injection of chloral hydrate solution (10%, 80 μL) combined with analgesia by intramuscular injection of ketamine (30 mg/kg weight) was performed on each mouse before blood collection and cervical dislocation.^33^ The left upper lobes were used for Western blot and left lower lobes for RT‐qPCR. The right lungs were used for pathology. The condition of the mice was assessed by measuring the change of body weight and the survival rate. The rate of weight change was determined using the equation: (weight at each time point − weight at zero hour)/weight at zero hour, and was analyzed between experimental groups on 1st day (24 hours), 6th day (144 hours), and 7th day (168 hours). Correlation between rate of weight change on 7th day and concentration of IL‐1β in serum was investigated.

### 2.6 Observation of pathological changes to the lungs

Fresh lungs were fixed in 4% paraformaldehyde, dehydrated, embedded in paraffin wax, and serially sectioned at 5 μm. Haematoxylin and eosin staining was performed and the slices were observed under the light microscope to assess pathological changes to the lung.

### 2.7 Quantitation of NLRP3, caspase‐1, and IL‐1β using RT‐qPCR

Total RNA was extracted from RAW264.7 cells and lungs using TRIzol Reagent (Takara, Japan). First‐strain complementary DNA was reverse‐transcribed from total RNA using the M‐MLV First‐Strain cDNA Synthesis Kit (Life Technologies Co, Ltd). Primers for all tested genes were designed and synthesized by Life Technologies Co, Ltd. The primers used in this analysis are shown in Table 1. RT‐qPCR was performed using an SYBR Select Master Mix qPCR Kit (Life Technologies Co, Ltd). The PCR protocol was as follow: 50°C for 2 minutes, 95°C for 2 minutes, then followed by 40 cycles of 95°C for 15 seconds, 60°C for 1 minute, then with an elongation step of 95°C for 15 seconds, 60°C for 1 minute, 95°C for 30 seconds, and 60°C for 15 seconds. β‐actin was used as the reference gene. The relative messenger RNA (mRNA) levels of NLRP3, caspase‐1, and IL‐1β were determined in triplicate using $2^{-\Delta \Delta C_{t}}$ method according to the equation: ΔC*t* =  C*t*_target gene_ − C*t*_β‐actin_, ΔΔC*t* = ΔC*t*_experimental group_ − ΔC*t*_control group_.^34^

**Table 1:** Primers used for RT‐qPCR

Genes	Sequence 5′‐3′
NLRP3	
Reverse	AACCAATGCGAGATCCTGACAAC
Forward	ACTGAAGCACCTGCTCTGCAAC
caspase‐1	
Reverse	AGTCACAAGACCAGGCATATTCT
Forward	TATAATGAAAGACGGCACACC
IL‐1β	
Reverse	TCACACACCAGCAGGTTATCATC
Forward	GAGCACCTTCTTTTCCTTCATCTT
β‐actin	
Reverse	CCAGTTGGTAACAATGCCATGT
Forward	GGCTGTATTCCCCTCCATCG

Abbreviations: IL, interleukin; NLRP3, nucleotide‐binding domain‐like receptor protein 3; RT‐qPCR, quantitative reverse transcription polymerase chain reaction.

### 2.8 Immunofluorescent staining of NLRP3 in macrophages

Immunofluorescent staining of NRLP3 in RAW264.7 macrophages from each group was performed with a primary monoclonal antibody against NLRP3 (Cell Signaling Technology, Danvers, MA) and a fluorescence‐conjugated secondary monoclonal antibody (R&D Systems, Inc, Minneapolis, MN). Nuclei were stained with Hoechst 33342 (Servicebio Technology Co, Ltd, Wuhan, China). Images were obtained for random fields under a laser scanning confocal microscope (Zeiss LSM710, Germany).

### 2.9 Measurement of the expression of NLRP3, caspase‐1, and cleaved caspase‐1 by Western blot

Proteins were extracted from RAW264.7 macrophages and fresh lung tissue using a Total Protein Extraction Kit (Nanjing KeyGenBiotech, Co, Ltd, China). Protein concentrations were determined by BCA Assay Kit (Nanjing KeyGenBiotech, Co, Ltd, China). After separating the proteins electrophoretically on a with 10% sodium dodecyl sulfate‐polyacrylamide gel electrophoresis, the total protein (30 μg/lane) was transferred onto a polyvinylidene fluoride (PVDF) membrane (Merck Millipore Co, Ltd, Germany). The PVDF membranes were blocked with Tris‐buffered saline Tween containing 5% skimmed milk and incubated with rabbit monoclonal glyceraldehyde 3‐phosphate dehydrogenase (GAPDH) (Jetway Biotech Co, Ltd, Guangzhou, China), NLRP3 (Cell Signaling Technology), caspase‐1 (Novus Biologicals, Centennial, CO), and cleaved caspase‐1 (Novus Biologicals) antibodies overnight at 4°C, and then incubated with a horseradish peroxidase‐labeled antirabbit secondary antibody (Jetway Biotech Co, Ltd, Guangzhou, China) for 1 hour. The blots were developed using an electrochemiluminescence color kit (Asbio Technology, Inc, Guangzhou, China), and densitometry was performed using Image J software. The relative protein expression levels of NLRP3, caspase‐1, and cleaved caspase‐1 were evaluated in triplicate using GAPDH as the internal standard.

### 2.10 Concentration of IL‐1β in the cell supernatant and serum measured by ELISA

The concentration of IL‐1β in the cell supernatant and serum was quantified using a Valukine ELISA Kit (R&D Systems, Inc). The ELISA Kit is specific for IL‐1β, and there is no cross‐reactivity with other cytokines. All samples were measured in triplicate. The concentration was determined by a standard curve using the mean absorbance values at 450 nm for each set of standards and samples.

### 2.11 Statistical analysis

The experimental data were processed and analyzed with the statistical software SPSS 20.0. All results are presented as mean ± standard deviation (*x* ± *s*). Statistical significance was analyzed by unpaired *t* test (for 2 means) or one‐way analysis of variance (for multiple groups). *LSD‐t* or *Dunnett T3* test was used for comparison between each two groups according to the homogeneity of variance. Correlation was investigated by Pearson's product‐moment correlation coefficient. Data were plotted using the GraphPad Prism software. A *P* value of <0.05 was considered to be statistically significant.

## 3 RESULTS

### 3.1 IAV infection activated the NLRP3 inflammasome, and MRSA infection secondary to IAV enhanced the expression of NLRP3 and caspase‐1 but decreased the expression of IL‐1β both in vitro and in vivo

**Figure 1:**
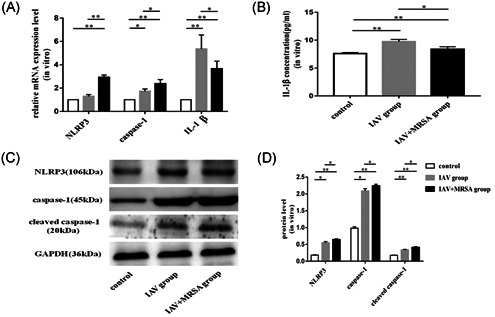
Expression levels of NLRP3, caspase‐1, cleaved caspase‐1, and IL‐1β in vitro. Murine macrophages were infected with IAV for 1 week in the IAV group, with IAV for 1 week, and coinfected with MRSA for 24 hours in the IAV + MRSA group. The control group received equivalent volumes of PBS. A, Relative mRNA expression levels of NLRP3, caspase‐1, and IL‐1β were measured by RT‐qPCR using the $2^{-\Delta \Delta C_{t}}$ method and β‐actin as the reference gene. B, Concentration of IL‐1β in the cell supernatant was assessed by ELISA. C and D, Relative protein expression of NLRP3, caspase‐1, and cleaved caspase‐1 were assessed by Western blot compared with the reference protein GAPDH. Protein level = densitometry of target protein/densitometry of GAPDH. *P < 0.05, **P < 0.01. ELISA, enzyme‐linked immunosorbent assay; GAPDH, glyceraldehyde 3‐phosphate dehydrogenase; IAV, influenza A virus; IL, interleukin; mRNA, messenger RNA; MRSA, methicillin resistant *Staphylococcus aureus*; NLRP3, nucleotide‐binding domain‐like receptor protein 3; PBS, phosphate‐buffered saline; RT‐qPCR, quantitative reverse transcription polymerase chain reaction

**Figure 2:**
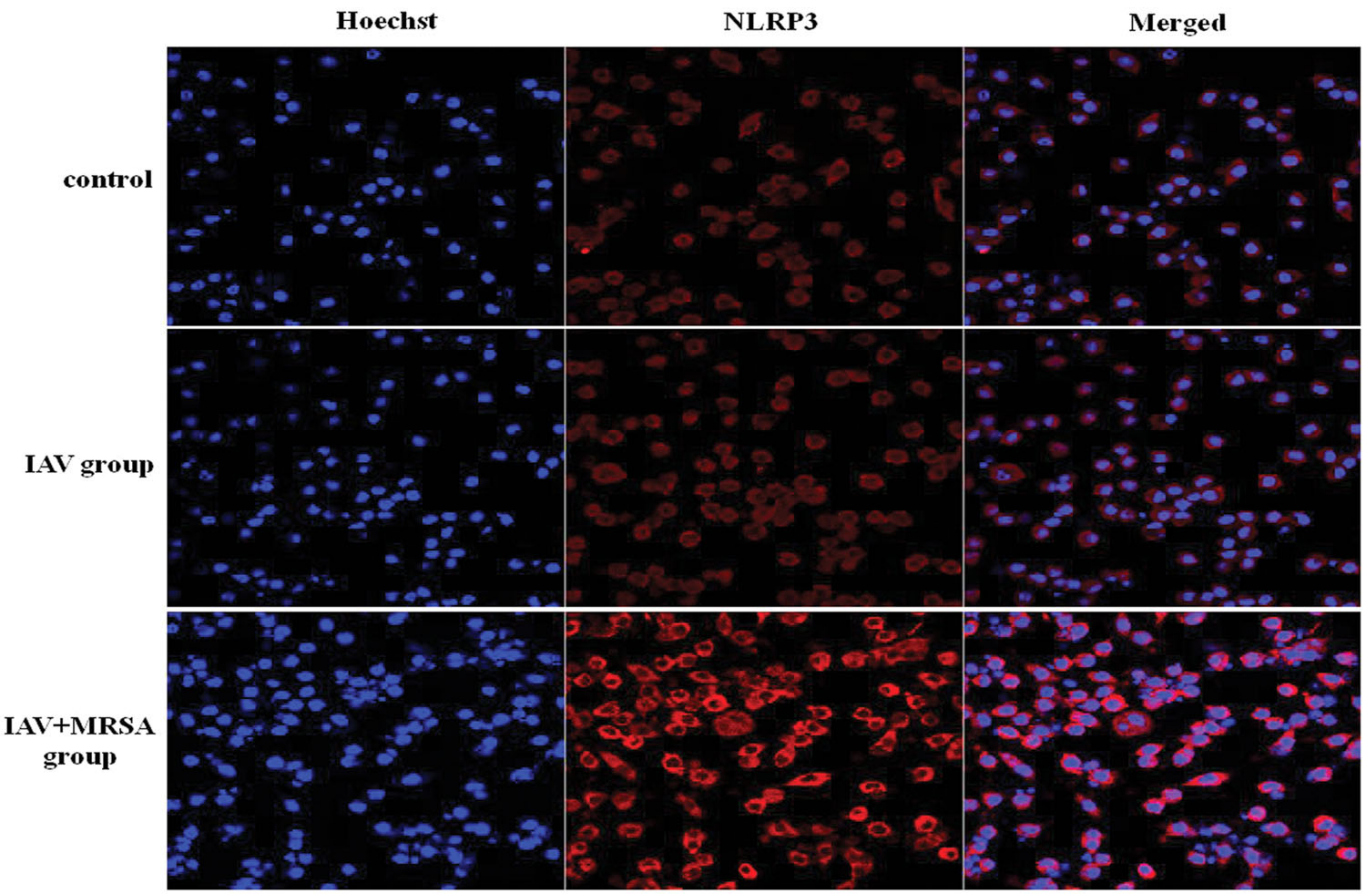
Immunofluorescent staining of NLRP3 in macrophages. Murine macrophages were infected with IAV for 1 week in the IAV group, with IAV for 1 week, and coinfected with MRSA for 24 hours in the IAV + MRSA group. The control group received equivalent volumes of PBS. The nucleus of murine macrophages was stained with Hoechst 33342 (blue, rank 1). NLRP3 was stained with a primary antibody and a fluorescence‐conjugated secondary antibody (red, rank 2), and these were merged (rank 3). Each group was processed under the same condition. Line 1 is the control group, line 2 is the IAV group, and line 3 is the IAV + MRSA group. IAV, influenza A virus; MRSA, methicillin‐resistant *staphylococcus aureus*; NLRP3, nucleotide‐binding domain‐like receptor protein 3; PBS, phosphate‐buffered saline

**Figure 3:**
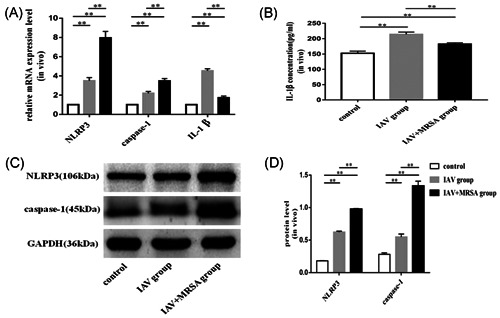
Expression levels of NLRP3, caspase‐1, and IL‐1β in vivo. Mice were infected intranasally with IAV for 1 week in the IAV group, with IAV for 1 week, and coinfected with MRSA for 24 hours in the IAV + MRSA group. The control group received equivalent volumes of PBS. A, Relative mRNA expression levels of NLRP3, caspase‐1, and IL‐1β were measured by RT‐qPCR using the 2^‐ΔΔCt^ method and β‐actin as the reference gene. B, Concentration of IL‐1β in serum was determined by ELISA. C and D, Relative protein expression of NLRP3 and caspase‐1 were assessed by Western blot compared with the reference protein GAPDH. Protein level = densitometry of target protein/densitometry of GAPDH. **P < 0.01. ELISA, enzyme‐linked immunosorbent assay; GAPDH, glyceraldehyde 3‐phosphate dehydrogenase; IAV, influenza A virus; IL, interleukin; mRNA, messenger RNA; MRSA, methicillin‐resistant *staphylococcus aureus*; NLRP3, nucleotide‐binding domain‐like receptor protein 3; RT‐qPCR, quantitative reverse transcription polymerase chain reaction

The mRNA levels of NLRP3, caspase‐1, and IL‐1β in the IAV and the IAV + MRSA groups were higher than those in the control group both in vitro and in vivo. And the mRNA levels of NLRP3 and caspase‐1 in the IAV + MRSA group were higher than those in the IAV group both in vitro and in vivo. However, the mRNA level of IL‐1β in the IAV + MRSA group was lower compared with the IAV group both in vitro and in vivo (Figures 1A and 3A). The fluorescence intensity of NLRP3 in macrophages increased in the IAV and the IAV + MRSA groups compared with that in the control group, and the fluorescence intensity of NLRP3 was higher in the IAV + MRSA group compared with that in the IAV group, which was consistent with the result of RT‐qPCR (Figure 2). Densitometry analysis of Western blots showed the same pattern of expression as the mRNA, with relative protein expression levels of NLRP3 and caspase‐1 increasing in the IAV and IAV + MRSA groups compared with those in the control group, with relative protein expression levels of NLRP3 and caspase‐1 in the IAV + MRSA group were higher compared with those in the IAV group both in vitro and in vivo (Figures 1C,D and 3C,D). Being in accordance with the increase of caspase‐1, upregulation of cleaved caspase‐1 was observed in vitro (Figure 1C,D). The concentration of IL‐1β in cell supernatant and serum both in the IAV and IAV + MRSA groups was higher than that in the control group. However, the concentration of IL‐1β in the cell supernatant and serum decreased in the IAV + MRSA group compared with that in the IAV group (Figures 1B and 3B). The concentration of IL‐1β in the cell supernatant decreased in IAV + MCC950 and IAV + MRSA + MCC950 groups compared with that in IAV and IAV + MRSA groups, respectively, but neither with statistical significance (Figure S1). These results collectively indicate that IAV infection for 1 week activates the NLRP3 inflammasome both in vitro and in vivo, and increased expression of NLRP3 and caspase‐1 are associated with MRSA infection secondary to IAV, but expression of IL‐1β is not related to NLRP3 and caspase‐1 during superinfection of MRSA and IAV. Besides, application of MCC950 on day 6 (144 hours) for 24 hours failed to decreased the concentration of IL‐1β further during IAV infection and MRSA superinfection in vitro.

### 3.2 Aggravated inflammatory pathology in the lung was associated with MRSA infection secondary to IAV in vivo

**Figure 4:**
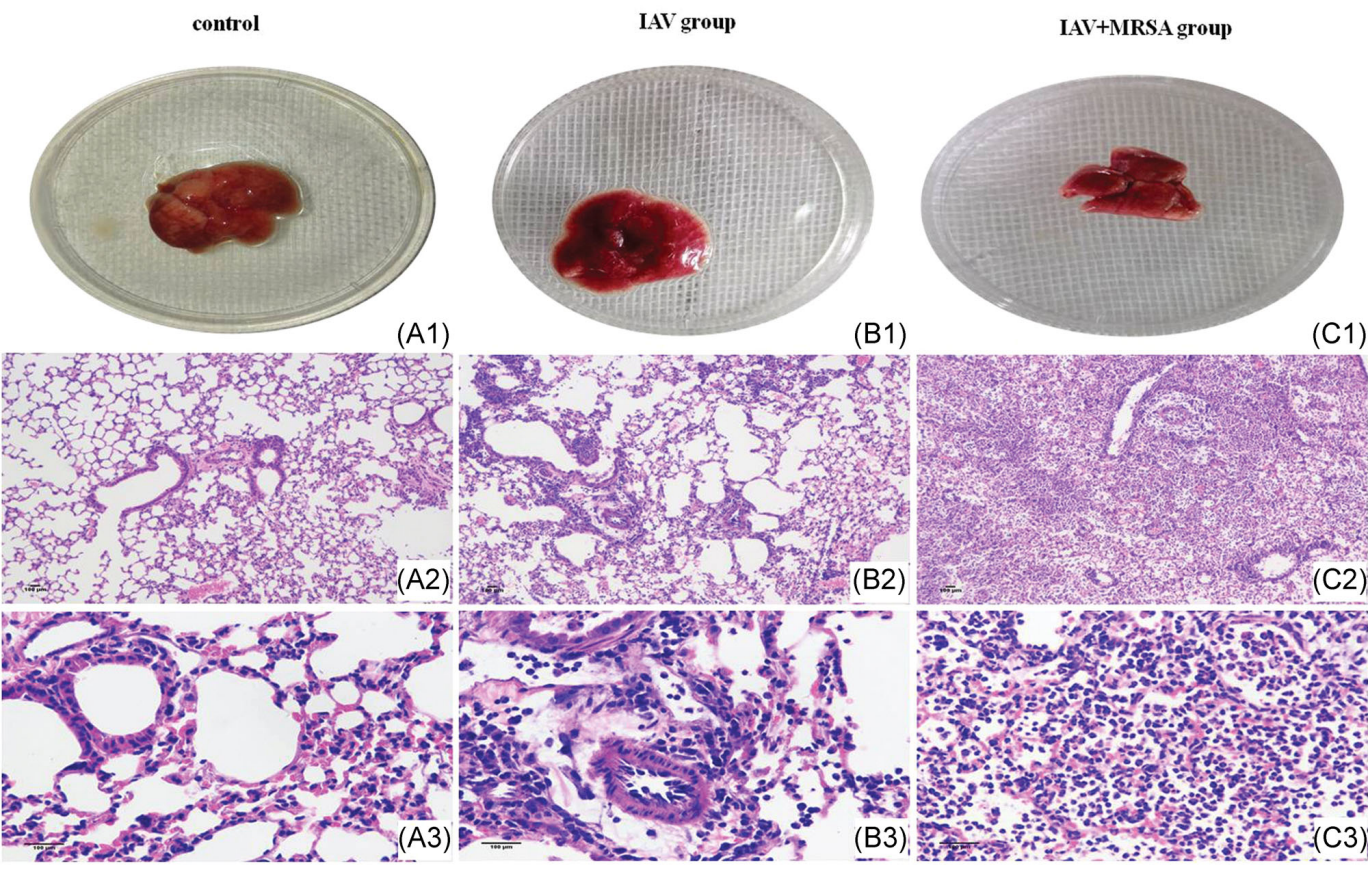
Pathology of the lungs in mouse model of pneumonia. Mice were infected intranasally with IAV for 1 week in the IAV group (B1, B2, and B3), with IAV for 1 week and then coinfected with MRSA for 24 hours in the IAV + MRSA group (C1, C2, and C3). Mice in the control group (A1, A2, and A3) received equivalent volumes of PBS intranasally. H&E staining. Line 1 shows the gross pathology of the lung. Lines 2 and 3 show the histopathology of the lung, with line 2 magnified ×100, and line 3 magnified ×400. Scale bars = 100 μm. H&E, hematoxylin and eosin; IAV, influenza A virus; MRSA, methicillin‐resistant *staphylococcus aureus*; PBS, phosphate‐buffered saline

The pathology of the lungs confirmed that pneumonia model caused by intranasal inoculation with IAV alone or with a secondary MRSA infection was successful. Gross pathology of the lung showed congestion in the IAV group (Figure 4B1), with more severe congestion in the lung of mice in the IAV + MRSA group (Figure 4C1), whereas the lung of mice in the control group was normal (Figure 4A1). Histopathology of the lungs in the control group revealed visible bronchioles and blood vessels, along with intact alveolar structure. The alveolar wall was thin, and there was no inflammatory infiltration in the alveolar cavity, nor in the interstitial substance (Figure 4A2,A3). In the IAV group, inflammatory cell infiltration and alveolar septum thickening were observed (Figure 4B2,B3). Whereas, more severe inflammatory damage was observed in the IAV + MRSA group. Infected lungs showed loss of alveolar architecture, infiltration of immune cells, vascular congestion, hemorrhage, bronchial obstruction, and even consolidation of lung parenchyma (Figure 4C2,C3). These results showed that more severe inflammatory pathology of the lungs was associated with MRSA infection secondary to IAV.

### 3.3 Increased weight loss of mice with secondary MRSA pneumonia was related to decreased concentration of IL‐1β in serum

**Figure 5:**
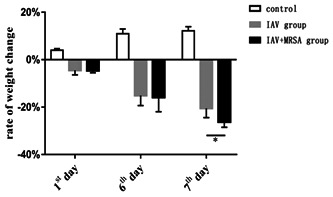
Changes in body weight during the experimental period. Mice were infected intranasally with IAV for 1 week in the IAV group, and with IAV for 1 week then coinfected with MRSA for 24 hours in the IAV + MRSA group. Mice in the control group received equivalent volumes of PBS intranasally. Rate of weight change = (weight at each time point − weight at zero hour)/weight at zero hour. First day is 24 hours, 6th day is 144 hours, and 7th day is 168 hours. *P < 0.05. IAV, influenza A virus; MRSA, methicillin resistant *staphylococcus aureus*; PBS, phosphate‐buffered saline

**Figure 6:**
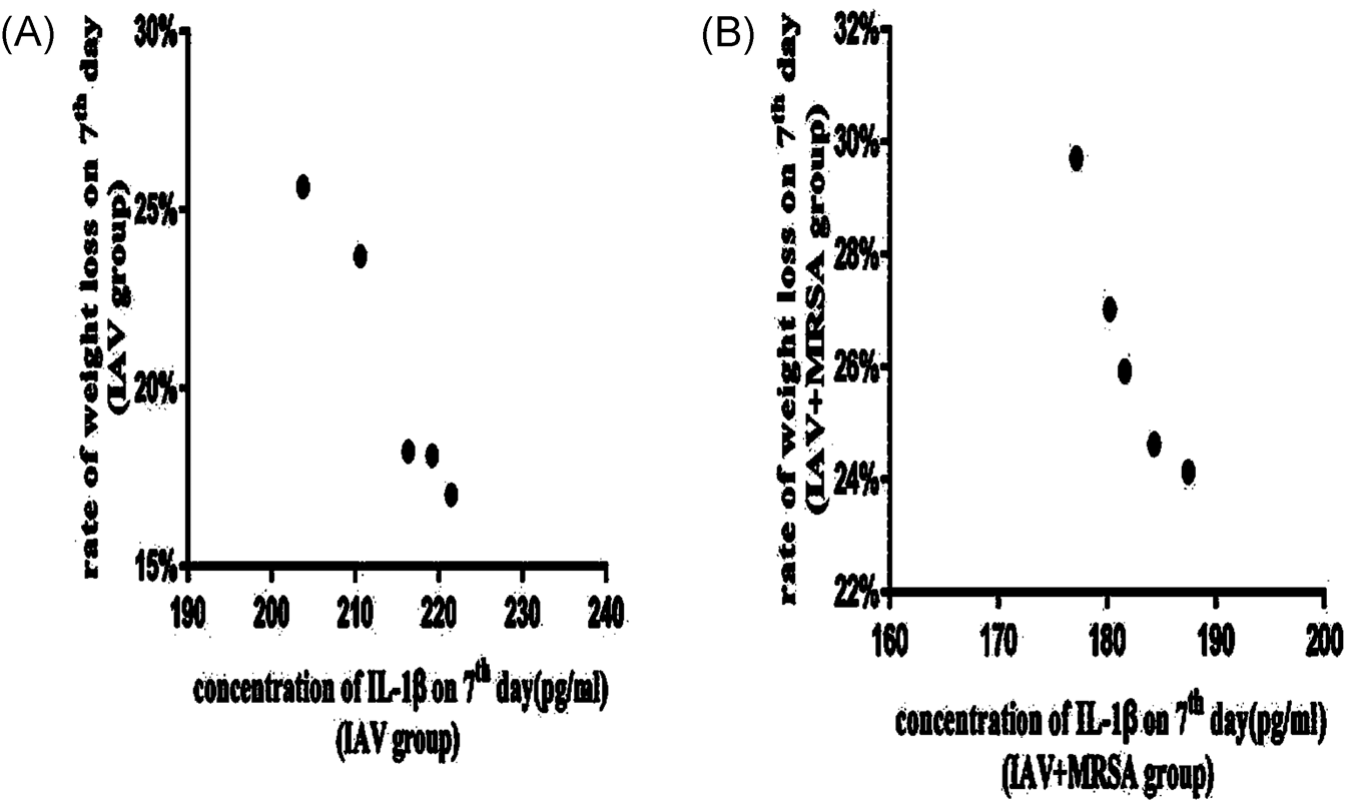
Scatter plots of correlation between rate of weight loss on 7th day and concentration of IL‐1β in serum on 7th day. Mice were infected intranasally with IAV for 1 week in the IAV group (A), and with IAV for one week then coinfected with MRSA for 24 hours in the IAV + MRSA group (B). Mice in the control group received equivalent volumes of PBS intranasally. Rate of weight loss on 7th day = (weight on 7th day − weight at zero hour)/weight at zero hour. Seventh day is 168 hours. IAV, influenza A virus; IL, interleukin; MRSA, methicillin‐resistant *Staphylococcus aureus*; PBS, phosphate‐buffered saline

All of the mice in the control, IAV, and IAV + MRSA groups survived for the duration of the experiment. Weight loss was used as a marker of severity and mortality. Mice in the control group increased in body weight at a normal rate, while mice in the IAV and IAV + MRSA groups had reduced food intake and activity, as well as significant weight loss during the experiment (Table 2). The condition of mice in the IAV + MRSA group deteriorated rapidly after coinfection with MRSA. During the 24 hours of superinfection, mice in the IAV + MRSA group lost more weight than mice in the IAV group (Figure 5). The rate of weight loss in the IAV and the IAV + MRSA groups on 7th day negatively correlated with concentration of IL‐1β in serum on 7th day in each group, respectively. Linear trend was found from the scatter plot (Figure 6A,B). Pearson's product‐moment correlation coefficient *r* = −.972 and *r* = −.953 (*P* < 0.01 and *P* < 0.05), respectively. These results showed that rate of weight loss in infected mice negatively correlated with concentration of IL‐1β in serum, which suggests that increased weight loss in MRSA pneumonia secondary to IAV infection was related to decreased concentration of IL‐1β in serum.

**Table 2:** Rate of weight change in mice

Timepoint, d	Rate of weight change (%)		
	Control	IAV group	IAV + MRSA group
First	3.99 ± 0.69	−4.48 ± 1.88	−4.57 ± 0.89
Sixth	10.96 ± 1.96	−15.12 ± 4.20	−15.86 ± 6.08
Seventh	12.16 ± 2.78	−20.53 ± 3.87	−26.29 ± 2.22
*F*	39.020	27.676	41.467
*P*	.000	.000	.000

*Note:* The rate of weight change = (weight at each time point − weight at zero hour)/weight at zero hour; 1st day was 24 h, 6th day was 144 h, and 7th day was 168 h. *P < 0.05.

Abbreviations: IAV, influenza A virus; MRSA, methicillin‐resistant *staphylococcus aureus*.

## 4 DISCUSSION

Both IAV and MRSA are common human pathogens. Superinfection with MRSA causes high mortality during influenza epidemics. AMs limit the replication of pathogens and eliminate infections in the lungs by producing cytokines such as IL‐1β.^35^ Numerous studies have identified the protective role of the NLRP3 inflammasome during IAV and MRSA infection via IL‐1β, which mediates inflammatory pathology and is associated with increased survival rate.^24^, ^36^ ^‐^ ^38^

Severe MRSA pneumonia usually occurs as a result of prior viral infection. And it is prone to occur at 5 to 7 days after the initial IAV infection.^39^, ^40^, ^41^, ^42^ However, the link between the NLRP3 inflammasome and aggravated pathology of the lungs during MRSA pneumonia secondary to IAV infection remains unclear. In the present study, the expression of the NLRP3 inflammasome both in vitro and in vivo, the pathology of the lungs, and the rate of weight change in mice were investigated in MRSA infection secondary to IAV.

In the present study, IAV infection for 1 week resulted in increased expression of NLRP3, caspase‐1, and IL‐1β both in RAW254.7 macrophages and in mice. Increased level of cleaved caspase‐1 in vitro was observed too. Pathology of the lungs showed inflammation in the IAV group. These results confirm that IAV infection activates NLRP3 inflammasome and induces inflammation, which is in agreement with previous reports.^43^, ^44^, ^45^, ^46^, ^47^

In the present study, we observed that the expression of IL‐1β decreased in MRSA infection secondary to IAV both in vitro and in vivo. And the rate of weight loss in infected mice negatively correlated with concentration of IL‐1β in serum. These results suggest that IL‐1β prevents the mice from weight loss during infection, and increased weight loss in MRSA pneumonia secondary to IAV infection is related to decreased concentration of IL‐1β in serum. As we know, IL‐1β is important for effective bacterial clearance during superinfection of *S. aureus* and IAV. Robinson et al^48^ found that a preceding IAV infection exacerbated *S. aureus* pneumonia by attenuating IL‐1β production. They also observed that IL‐1β was essential for bacterial clearance during superinfection of *S. aureus* and IAV.^23^ Therefore, we infer that superinfection with MRSA reduces expression of IL‐1β someway, and decreased expression of IL‐1β impairs the host immunity and leads to aggravated pneumonia. More severe inflammation in pathology of the lungs during the superinfection with MRSA supports the inference.

As to the divergent expression of NLRP3 inflammasome and IL‐1β. Enhanced expression of NLRP3, caspase‐1, and cleaved caspase‐1 are associated with MRSA infection secondary to IAV. However, both transcription and secretion of IL‐1β decreased with MRSA superinfection. And MCC950 failed to decreased the concentration of IL‐1β further during MRSA superinfection in vitro. These results suggest that superinfection with MRSA upregulates the expression of NLRP3 and caspase‐1 but interferes with the upstream signaling of IL‐1β somehow. Excessive inflammation in MRSA infection secondary to IAV may be related to another mechanism independent of IL‐1β. Some studies implied clues to potential mechanism. Gomes de Morais et al^30^ found that deregulation of the expression of TLR and NLRP3 receptors may induce extensive tissue injury and favors the longevity and spread of MRSA. Kremserova and Nauseef^49^ found that neutrophils induce inflammation through a mechanism independent of NLRP3 and IL‐1β in MRSA infection. While van Kruchten et al^28^ found that superinfection with *S. aureus* caused more severe damage by triggering a shift from influenza‐induced apoptosis to necrotic cell death. The details of this mechanism should be the subject of further study, as it is likely that effective treatment is linked to this inflammatory response.

The present study supports previous findings that immunotherapy targeting the NLRP3 inflammasome or IL‐1β signaling pathway may reduce the severity and improve the clinical outcomes in MRSA pneumonia secondary to IAV infection.^50^ It may also provide potential insights into the prevention and treatment of other viral epidemics, such as coronavirus disease 2019.

In conclusion, we found that the upregulation of the NLRP3 inflammasome was associated with MRSA infection secondary to IAV, but that MRSA superinfection resulted in a decrease in the expression of IL‐1β someway, by which impair the host immunity and lead to aggravated pneumonia. Further studies are required to fully understand the effects of NLRP3 inhibition and to identify the mechanism by which IL‐1β expression is decreased during MRSA superinfection. It is expected that these findings will provide vital insights into potential immunotherapy targets for treating MRSA pneumonia secondary to IAV infection.

## AUTHOR CONTRIBUTIONS

YS, XS, and BW conceived and designed the experiments. YS and XS performed the experiments and analyzed the data, then wrote the paper. JLi, JLu, and JB provided advice and assistance. JC carried out the examination of pathology.

## Supporting information

Supplementary information

Supplementary information

## Data Availability

The data that support the findings of this study are available from the corresponding author upon reasonable request.
