# Supplementary figures and images for: Aggravated MRSA pneumonia secondary to influenza A virus infection is derived from decreased expression of IL‐1β
Source: J Med Virol. 2020 Sep 16;92(12):3047–56. doi: 10.1002/jmv.26329 (PMC7692898; doi:10.1002/jmv.26329)

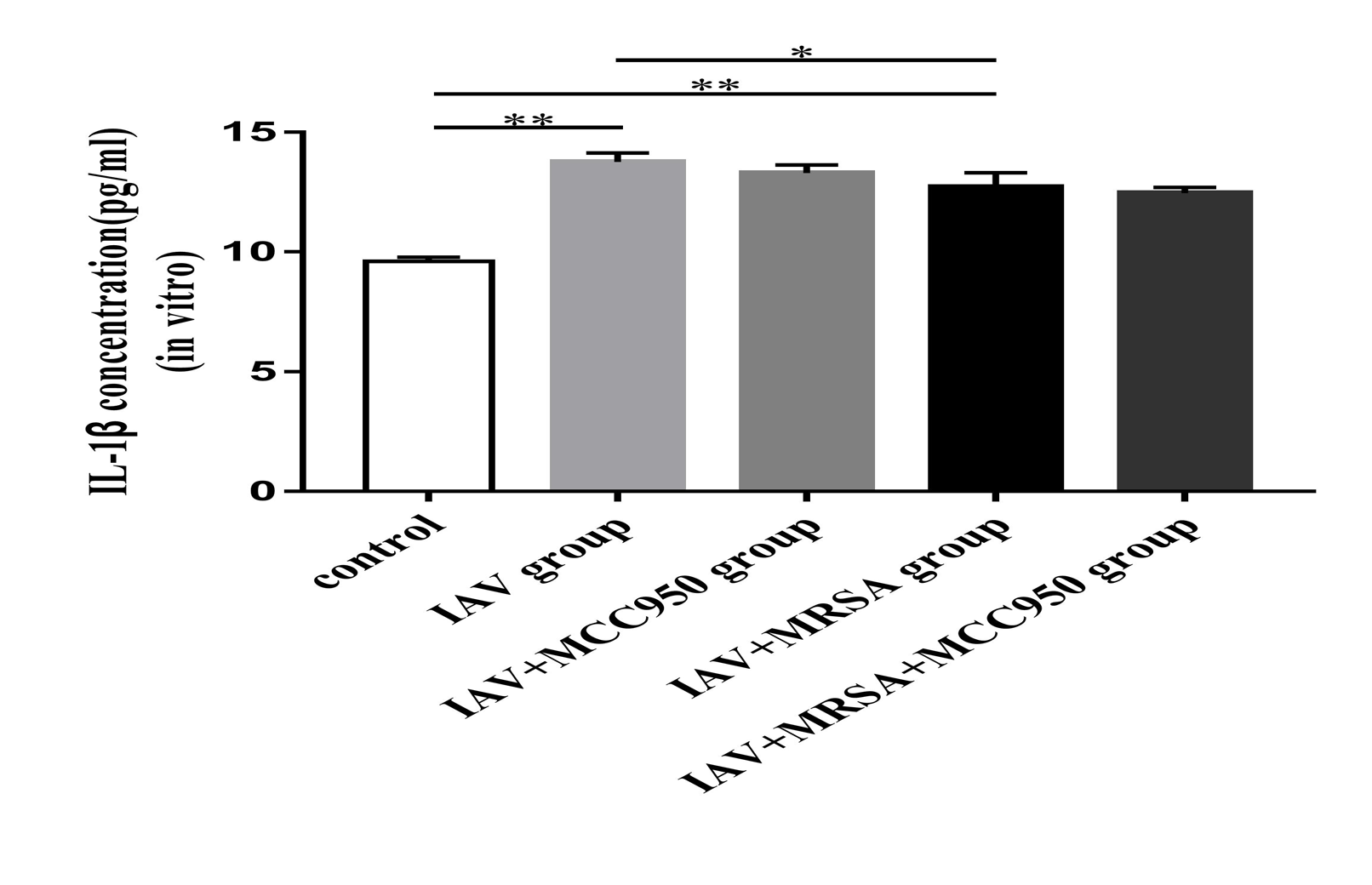

Supplement: Supplementary file 1 — Supplementary information [file JMV-92-3047-s001.tif]
