# Supplementary material for: Aggravated MRSA pneumonia secondary to influenza A virus infection is derived from decreased expression of IL‐1β
Source: J Med Virol. 2020 Sep 16;92(12):3047–56. doi: 10.1002/jmv.26329 (PMC7692898; doi:10.1002/jmv.26329)
Supplement: Supplementary file 2 — Supplementary information [file JMV-92-3047-s002.docx]

**Supplemental Fig. 1** Concentration of IL-1β in the cell supernatant

Murine macrophages were infected with IAV for one week in the IAV and IAV+MCC950 groups, with IAV for one week and co-infected with MRSA for 24 hours in the IAV+MRSA, IAV+MRSA+MCC950 groups. 7.5μM MCC950 was applied to the IAV+MCC950 and IAV+MRSA+MCC950 group on day 6 (144h). The same volume of PBS was added to the other groups every time when stimulation was carried out. * p<0.05, ** p<0.01.
